# Use of electronic medical records to describe the prevalence of allergic diseases in Canada

**DOI:** 10.1186/s13223-021-00580-z

**Published:** 2021-08-18

**Authors:** Alexander G. Singer, Leanne Kosowan, Nerissa Nankissoor, Ryan Phung, Jennifer L. P. Protudjer, Elissa M. Abrams

**Affiliations:** 1 Department of Family Medicine, University of Manitoba, Winnipeg, Canada; 2 University College Dublin School of Medicine, Belfield, Dublin, Ireland; 3 Department of Pediatrics and Child Health, University of Manitoba, Winnipeg, Canada; 4 Department of Pediatrics, Section of Allergy and Clinical Immunology, University of Manitoba, FE125-685 William Avenue, Winnipeg, MB R3E 0Z2 Canada

**Keywords:** Allergy prevalence, Primary care

## Abstract

**Background:**

Leveraging the data management resources of the Canadian Primary Care Sentinel Surveillance Network (CPCSSN) is a viable approach for describing the prevalence of allergic disease documented in primary care settings.

**Methods:**

The dataset used for this study was inclusive of data from EMR initiation up to Dec 31st 2018. The sample included 1235 primary care providers representing 1,556,472 patients across Canada.

**Results:**

In total, there were 536,005 patients with a documented allergy that fit into one of the 10 suggested categories. The allergy table includes 718,032 distinct entries representing 564,242 unique patients, which is 36.3% of the patients within the CPCSSN repository. The most common allergies recorded were drug allergy (39.0%), beta-lactam allergy (14.4%), environmental allergy (11.0%), and food allergy (8.0%). Anticipated upcoming studies include physician-documented drug allergy with a focus on beta-lactam allergy, as well as stinging insect allergy, among others. To our knowledge, these will also be the first such prevalence studies of primary care physician-documented allergic disease done in Canada.

**Interpretation:**

The CPCSSN dataset represents electronic medical records from 1.5 million patients across Canada including documentation of allergic diseases. This dataset provides a national representative population to describe and characterize Canadian patients with common allergic conditions. This robust dataset provides the opportunity for health surveillance, and in particular data to explore the impact of allergic disease on primary care practice.

**Trial registration:**

Not applicable.

## Introduction

Disease prevalence rates can estimate the burden of disease, highlight research priorities, direct guidelines and medical policy, inform healthcare economic models, and provide a baseline for interventional research (by providing baseline risk in a population) [1]. Prevalence rates for allergic conditions can be determined by various means including self-report, or medical record data. Prevalence of common allergic conditions can vary significantly between studies, with self-reported allergy often higher than diagnosed allergy [2].

Administrative data in Canada are typically captured for the primary purpose of remuneration. Administrative claims data used for remuneration includes diagnostic codes held in provincial data repositories that record the primary condition managed at each patient appointment. Conversely, clinical datasets such as those from Electronic Medical Records (EMRs) provide a more comprehensive health record inclusive of a patient’s history including details such as diagnoses, prescriptions, visit details and biometric measures. It has been noted that EMR data, in contrast to administrative claims data, provides more expedient information and allows a better glimpse of the clinically pertinent results of the medical encounter [3, 4]. With this understanding, primary care physician documentation present an opportunity to estimate the prevalence of allergic conditions.”

The majority of medical care within Canada is provided in primary care settings. Leveraging the data management resources of the Canadian Primary Care Sentinel Surveillance Network (CPCSSN) is a viable approach for describing the prevalence of allergic disease documented in primary care settings. CPCSSN extracts EMR data from participating primary care providers across Canada, such as de-identified medication tables, billing tables, health conditions tables, etc. The repository has been shown to be representative of the Canadian population with age and sex adjustment [5].

CPCSSN has developed and refined processes for cleaning and preparing data for secondary quality improvement, research and surveillance activities. Our goal is to describe the CPCSSN data extraction process, and how it will be used to capture primary care clinician-documented allergic disease within Canada. Our group has already reported this for food allergy, and to our knowledge this was the first primary care clinician-documented allergic prevalence study in Canada [6].

## Methods: data set extraction

The dataset used for this study was inclusive of data from EMR initiation of each provider to Dec 31st 2018. The sample included 1235 primary care providers representing 1,556,472 patients across Canada. Seven provinces (i.e. Ontario, Alberta, Nova Scotia, British Columbia, Manitoba, Newfoundland and Quebec) and 11 EMR vendors were represented in this data extract. The largest represented EMR vendors were Accuro, Practice Solutions, Nightingale, Wolf, Med Access and OSCAR.

**Fig. 1:**
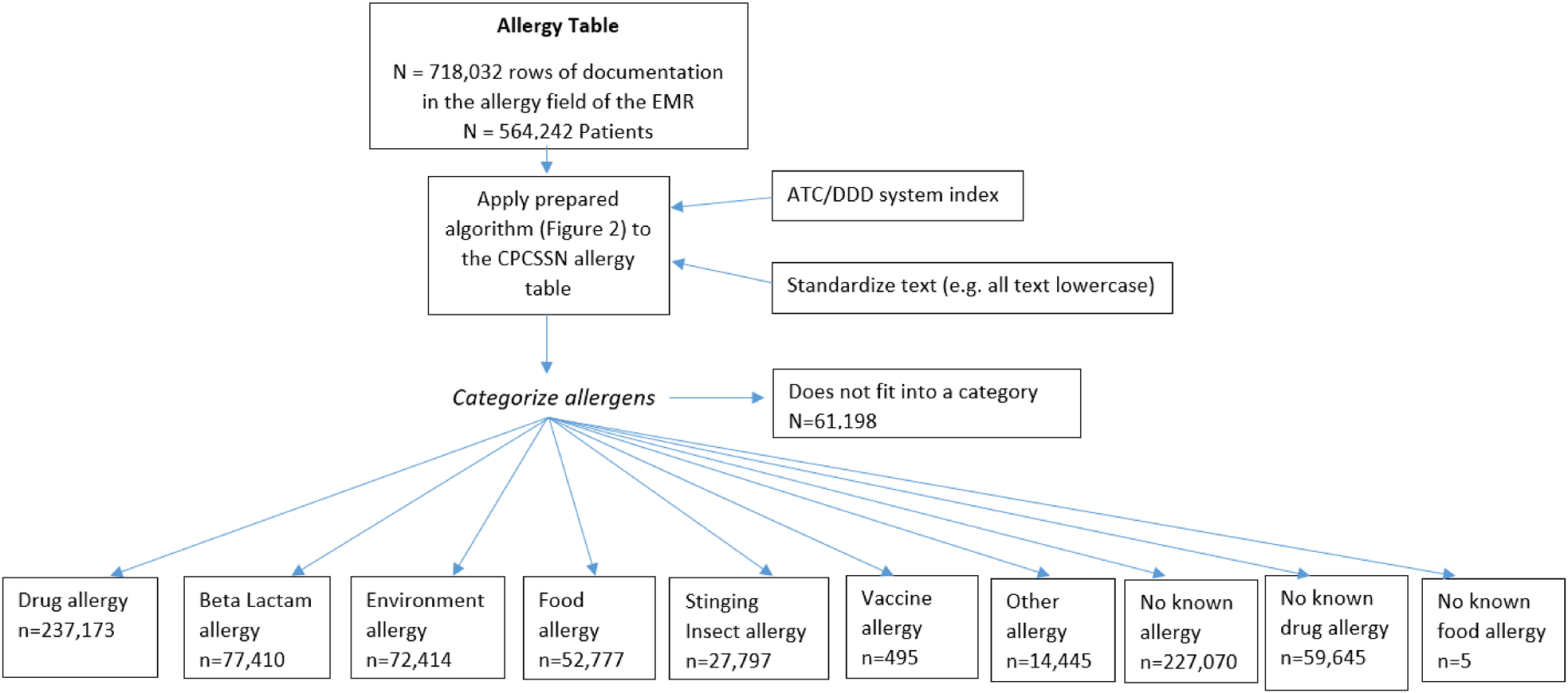
Documentation of allergy categories in the CPCSSN allergy table

The allergy table within CPCSSN included 718,032 distinct entries representing 564,242 unique patients, which is 36.3% of the patients within the CPCSSN repository (Fig. 1). The allergy table included a semi-structured text field for the allergen, and a field for possible drug code (for a medication allergy). Original text input by the clinician was cleaned and processed to create a calculated field. This included preprocessing stages to prepare the data for categorization (e.g. removing stop words and punctuation), and assigning an ATC code to medication names using the ATC/DDD system index. A chart review was conducted to assign a category to free-text allergy entries. Categories of common allergies were: drug allergy (overall), beta-lactam allergy (specifically), environmental allergy, food allergy, stinging insect allergy, and vaccine allergy.

**Fig. 2:**
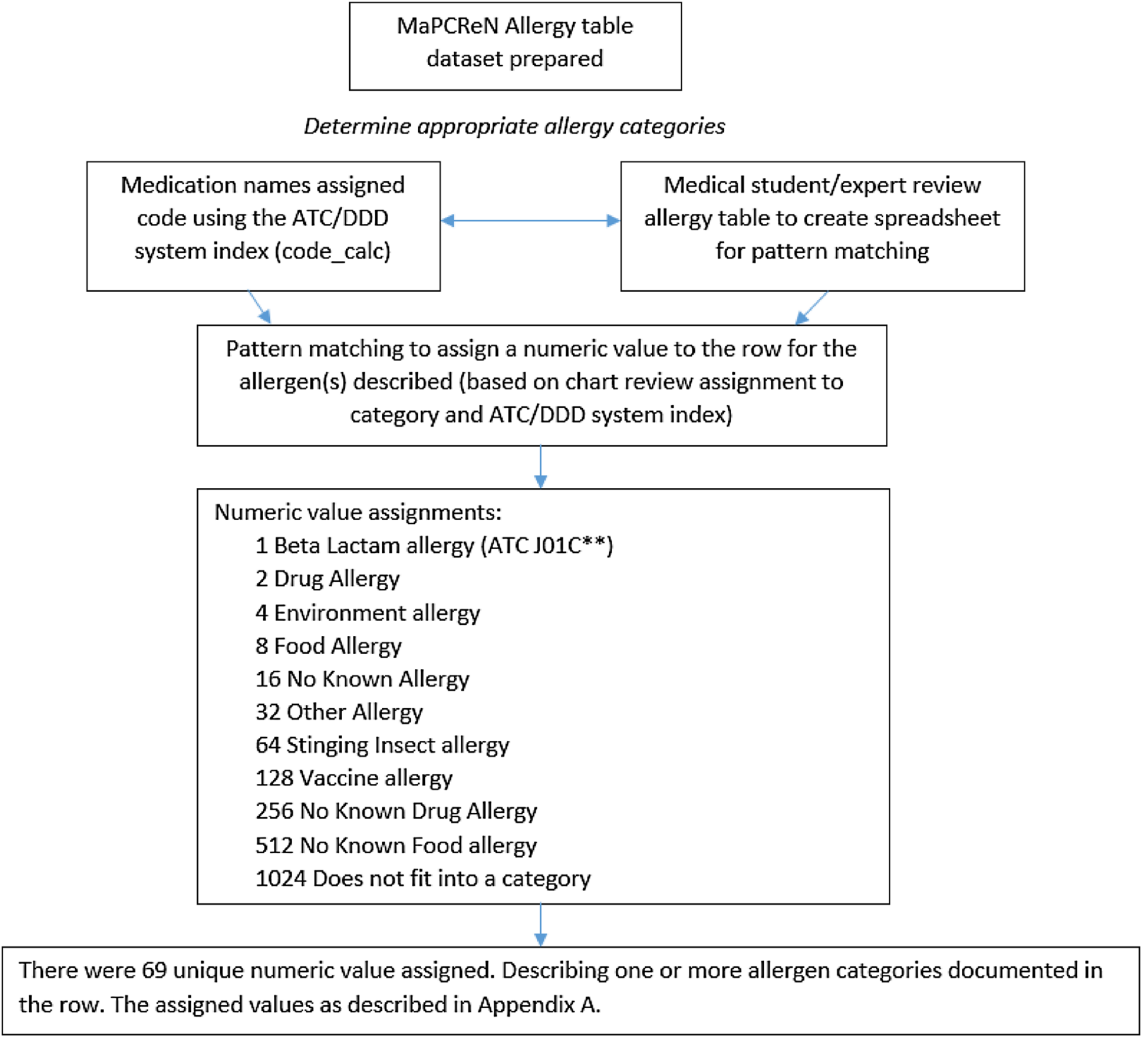
Building an algorithm to categorize the allergens documented in the CPCSSN allergy table

Using available free text from the CPCSSN allergy table unique terms (key words, known abbreviations, common incorrectly spelled terms, etc.) were labeled as being associated with one of the pre-defined allergy categories (Fig. 2). The ‘other’ category was created to capture entries documenting allergens not captured within the predefined categories (such as ‘red dye allergy’).

Algorithms were developed within SQL to match all occurrences of the labelled terms to a specific category. Using pattern matching SQL assigned a numeric value to each of the allergy categories, summing the numeric values if more than one allergy category was presented in a single field. Pattern matching resulted in 69 unique numeric values each representing one or more documented allergies. The processing algorithm did not categorize 61,198 entries because they represented non-allergy values including reactions or investigations without an allergen mentioned (Fig. 1).

### Described and anticipated outcomes

Figure 1 lists the number of entries within each allergy category. In total, there were 536,005 patients with a documented allergy that fit into one of the 10 suggested categories. The most common allergies recorded were drug allergy (39.0%), beta-lactam allergy (14.4%), environmental allergy (11.0%), and food allergy (8.0%) (Table 1). Thus far, our group has described the physician-documented prevalence of pediatric food allergy based on this dataset [6]. Anticipated upcoming studies include physician documented drug allergy with a focus on beta-lactam allergy, and stinging insect allergy, among others. To our knowledge, these will also be the first such prevalence studies done in North America.

**Table 1:** Documentation of allergy in the CPCSSN allergy table

Allergy category	Number of observations	Number of patients
No known allergy	227,070 (34.6%)	227,050 (42.4%)
Drug	237,173 (36.1%)	209,028 (39.0%)
Beta-lactam	77,410 (11.8%)	77,353 (14.4%)
Environment	72,414 (11.0%)	62,164 (11.6%)
No known drug allergy	59,645 (9.1%)	53,196 (9.9%)
Food	52,777 (8.0%)	48,822 (9.1%)
Stinging insect	27,797 (4.2%)	26,397 (4.9%)
Other	14,445 (2.2%)	14,025 (2.6%)
Vaccine	495 (0.1%)	495 (0.1%)
No known food allergy	5 (0.0%)	5 (0.0%)
Total	656,834	536,005

## Discussion/conclusion

The CPCSSN dataset provides a prevalence estimate for common physician documented allergic diseases, among a representative sample of 1.5 million patients across Canada [5]. In addition CPCSSN provides an avenue to describe and characterize Canadian patients with common allergic conditions including associated comorbidities (e.g. atopic conditions) [6]. Studies of allergic disease prevalence within North America have largely focused on self-report [2]. This comprehensive dataset can inform health surveillance exercises aimed at understanding allergic disease prevalence rates in primary care and their relationship to health service utilization and outcomes.

The dataset relies on primary care provider documentation within the EMR, which does have the potential to either overestimate or underestimate the ‘true’ prevalence. The prevalence may be overestimated as this algorithm was not designed to detect the results of confirmatory testing or consultation reports. Physician reports of some allergies, such as drug allergy, have been shown to overestimate true prevalence [7]. In Canada, confirmatory testing and consultation reports are held provincially, future work should explore the agreement between documentation and true allergy prevalence. In addition, while associations with other comorbidities can be determined, a causal association cannot be elucidated.

In conclusion, we describe a novel approach to the description of allergy prevalence within Canada. While there are strengths and limitations to each approach used to describing allergy prevalence, our approach provides a unique lens through which to describe the burden of allergic disease within Canada, and its associated comorbidities.

## Data Availability

The datasets generated and/or analysed during the current study are not publicly available due to the confidential nature of data governed by the PHIA legislation.

## Abbreviations

CPCSSN: Canadian Primary Care Sentinel Surveillance Network
EMR: Electronic Medical Record
